# Structural basis for promotion of duodenal iron absorption by enteric ferric reductase with ascorbate

**DOI:** 10.1038/s42003-018-0121-8

**Published:** 2018-08-17

**Authors:** Menega Ganasen, Hiromi Togashi, Hanae Takeda, Honami Asakura, Takehiko Tosha, Keitaro Yamashita, Kunio Hirata, Yuko Nariai, Takeshi Urano, Xiaojing Yuan, Iqbal Hamza, A. Grant Mauk, Yoshitsugu Shiro, Hiroshi Sugimoto, Hitomi Sawai

**Affiliations:** 1 Graduate School of Life Science, University of Hyogo, 3-2-1 Kouto, Kamigori, Ako, Hyogo 678-1297 Japan; 2 RIKEN SPring-8 Center, 1-1-1 Kouto, Sayo, Hyogo 679-5148 Japan; 3 Department of Biochemistry, Shimane University School of Medicine, 89-1 Enya, Izumo, Shimane 693-8501 Japan; 4 Department of Animal and Avian Sciences, University of Maryland, 8127 Regents Drive, College Park, MD 20742 USA; 5 Department of Biochemistry and Molecular Biology and Centre for Blood Research, University of British Columbia, 2350 Health Sciences Mall, Vancouver, BC V6T 1Z3 Canada

## Abstract

Dietary iron absorption is regulated by duodenal cytochrome *b* (Dcytb), an integral membrane protein that catalyzes reduction of nonheme Fe^3+^ by electron transfer from ascorbate across the membrane. This step is essential to enable iron uptake by the divalent metal transporter. Here we report the crystallographic structures of human Dcytb and its complex with ascorbate and Zn^2+^. Each monomer of the homodimeric protein possesses cytoplasmic and apical heme groups, as well as cytoplasmic and apical ascorbate-binding sites located adjacent to each heme. Zn^2+^ coordinates to two hydroxyl groups of the apical ascorbate and to a histidine residue. Biochemical analysis indicates that Fe^3+^ competes with Zn^2+^ for this binding site. These results provide a structural basis for the mechanism by which Fe^3+^ uptake is promoted by reducing agents and should facilitate structure-based development of improved agents for absorption of orally administered iron.

## Abstract (web summary)

Ganasen et al. report the crystallographic structures of human duodenal cytochrome *b* and its complex with ascorbate and Zn^2+^. This study provides mechanistic insights into how reducing agents promote the uptake of orally administered iron and may facilitate the development of such interventions.

## Introduction

Iron was believed to have therapeutic value as early as 1500 BCE, though few early uses for such treatment are now regarded as rational. Modern therapeutic use of orally administered iron was introduced by the English physician Thomas Sydenham (1624−1689) in the seventeenth century for the treatment of chlorosis (now known as iron deficiency anemia) with “iron tonic” (iron filings solubilized by tannins in wine), several years before the presence of iron in blood was demonstrated (Lemery and Geoffroy, 1713) and before consumption of iron was shown to increase the iron content of blood (Menghini, 1746). The extensive history of iron in medical practice has been reviewed elsewhere^1,2^. Today, iron is recognized as an essential nutrient that is required for oxygen transport, energy production, and synthesis and metabolism of many bioactive compounds in all living organisms^3^. Despite this long-standing medical use of iron, iron deficiency continues to affect 30% of the world population, is the most severe and widespread nutritional deficiency disorder, and remains the only nutritional deficiency disorder in industrialized countries^4,5^. On the other hand, iron overload causes accumulation of iron in various tissues and can result in cirrhosis^6^, neurodegenerative and cardiovascular diseases (cardiomyopathy, etc.)^7,8^ that result from formation of reactive oxygen species through Fenton or Haber−Weiss reactions^9^. These pathological processes are normally prevented through strict regulation of iron uptake, storage and distribution because there is no regulated route of iron excretion.

Iron deficiency anemia has many etiologies, but the primary causes are the difficulty of iron absorption in the gut, the loss of blood iron (primarily from menses), and the increased need for iron during pregnancy. The average adult possesses 50 mg per kg of body weight of iron that is obtained from the diet (1–2 mg per day) in two forms, heme (Fe-protoporphyrin IX) and nonheme iron. Heme iron is derived from the hemoglobin and myoglobin present in red meat and is more readily absorbed (12–25% bioavailable) than nonheme iron (<5% bioavailable)^10^, which is typically derived from plants. Nonheme iron occurs primarily as Fe^3+^, which occurs in insoluble forms at enteric pH unless bound to soluble chelators.

Absorption of nonheme iron has long been known to be enhanced by reducing agents such as ascorbate^11^. With the discovery of the enteric Fe^3+^ reductase Dcytb^12^ and the divalent metal transporter DMT-1^13^, the proteins responsible for uptake of dietary nonheme (elemental) iron are now known. Dcytb is an iron-regulated Fe^3+^ reductase that was first identified in the duodenal brush border of mice with systemic iron deficiency^12^. Since DMT-1 favors the absorption of divalent metal including Fe^2+^^13^, the reduction of Fe^3+^ to Fe^2+^ by Dcytb in the duodenum is essential for effective intestinal iron absorption. Dcytb utilizes ascorbate in cytoplasm as an electron donor to reduce apical Fe^3+^. Notably, the expression and activity of mucosal Dcytb are closely associated with chronic anemia and hypoxia^14^. Human enteric ferric reductase Dcytb is a member of the cytochrome *b*_561_ (Cytb_561_) protein family, all of which are ascorbate-dependent oxidoreductases that are homo-dimeric, integral membrane proteins in which each monomer possesses six transmembrane helices and two heme b groups^15,16^. All members of this family use cytoplasmic ascorbate as an electron donor for the reduction of a substrate at the noncytoplasmic side of the enzyme in which the two heme b groups mediate intra-molecular electron-transfer reaction across the membrane. Cytb_561_ proteins are identified not only in animals but also in plants. The plant Cytb_561_ is known to be involved in ascorbate recycling. The role of plant Cytb_561_ as Fe^3+^ reductase has not been fully established, because another family of enzymes, the ferric reductase oxidase family, is involved in cellular iron uptake^17,18^. To date, detailed mechanistic understanding of how these proteins perform their functions has remained elusive. The three-dimensional structure of Dcytb reported in the current study addresses this deficiency by providing structural insight into the mechanism by which ascorbate reduction of Fe^3+^ is catalyzed to enable iron absorption.

## Results

### Structural analysis of Dcytb

Recombinant human Dcytb was expressed in *Escherichia coli* and purified by nickel-affinity and size exclusion chromatography. The purified protein was stable and mono-disperse. The electronic absorption spectrum is characteristic of the spectrum of bis-His coordinated hemoproteins with α- and β-bands observed at 531 and 561 nm, respectively, for the ferrous form (Supplementary Fig. 1). These spectra were essentially identical to those previously reported for Dcytb expressed in Sf9 cells^19^ or *E*. *coli*.

This recombinant Dcytb was crystallized in the lipidic cubic phase (LCP) in the absence of substrate (ascorbate or metal ion). The resulting crystal belongs to the *C*2 space group and allowed collection of X-ray diffraction data to a resolution of 2.6 Å. The structure derived from these data was determined by molecular replacement on the basis of the structure reported for *Arabidopsis thaliana* Cytochrome *b*_561_ (*At*Cytb_561_)^20^, which exhibits 36% sequence identity with human Dcytb. Dcytb and *At*Cytb_561_ belong to the cytochrome *b*_561_ family, members of which have two heme cofactors per monomer and act as ascorbate-dependent oxidoreductases^15^.

**Fig. 1:**
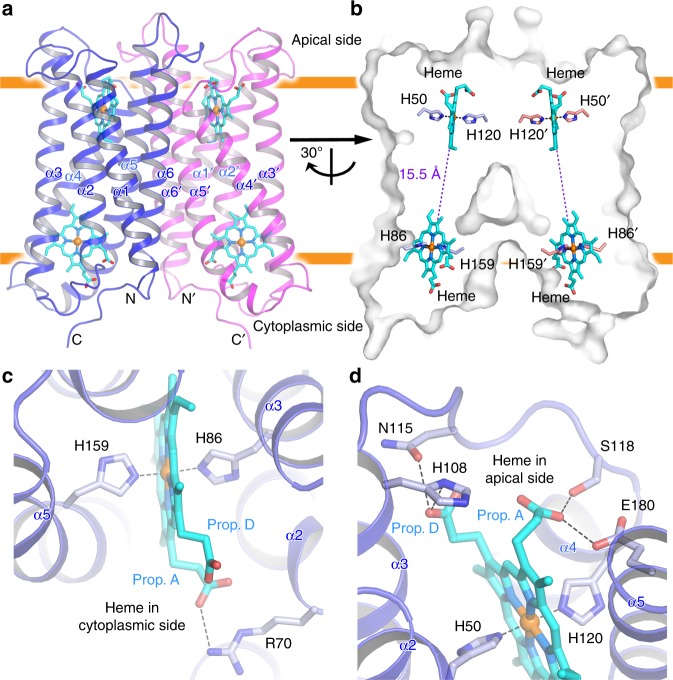
Overall structure of human Dcytb. **a** Ribbon representation of the Dcytb homodimer. Each monomer consists of six α-helices (α1−α6). Both N- and C- termini are located on the cytoplasmic side. Each monomer contains two heme b molecules, coordinated by four highly conserved His residues in a six-coordinate low spin form. **b** The edge-to-edge distance between two heme molecules is 15.5 Å. **c** The environment of the heme bound to the cytoplasmic side of Dcytb is shown. **d** The environment of the heme bound to the apical side of Dcytb is shown

The overall structure of human Dcytb is a homodimer in which each monomer possesses six transmembrane α-helices. The N- and C-termini are located on the cytoplasmic side of the protein (Fig. 1a). The Dcytb dimer exhibits crystallographic twofold symmetry in which the monomers interact through hydrophobic contacts involving transmembrane helices α5 and α6 on the cytoplasmic side and helices α4, α5 and α6 on the apical side (Supplementary Fig. 2). Although full-length Dcytb (residues 1–286) was used for crystallization, the atomic model includes only residues 6–230 because the electron density map for the five N-terminal residues and the 56 C-terminal residues was disordered.

The two heme b groups of each Dcytb monomer are sandwiched between four central transmembrane helices (α2−α5) at the apical (or luminal) and cytoplasmic ends of the structure and are referred to as such. As expected from the electronic absorption spectrum, each heme is hexacoordinate with H86 and H159 coordinating the iron of the cytoplasmic heme and H50 and H120 coordinating the apical heme iron (Fig. 1b). All four of these ligands are highly conserved in the Cytb_561_ family. The A-propionate of the cytoplasmic heme forms a salt-bridge with the side chain of R70 from α2, and the corresponding D-propionate is exposed to the solvent (Fig. 1c). The A-propionate of the apical heme forms a hydrogen-bond with the side chains of E180 from α5 and S118 from α4, and the D-propionate is hydrogen bonded with the side chain of N115 from α3 (Fig. 1d). The closest edge-to-edge distance between the two heme groups within a monomer is 15.5 Å (Fig. 1b).

To characterize the structural basis for ascorbate and metal ion binding to Dcytb, we cocrystallized Dcytb in the presence and absence of ascorbate with and without metal ions (Fe^3+^, Cu^2+^, and Zn^2+^) or other compounds such as ferric ammonium citrate and hydroxypyridone. Only soaking crystals with Zn^2+^ and ascorbate produced crystals that diffracted X-rays well. Although Fe^3+^ and Cu^2+^ are substrates for Dcytb, these metal ions are spontaneously reduced by ascorbate, and reduction affects the affinity for the protein or damages the crystal. Zn^2+^ is not redox active and is generally known to bind proteins in a manner similar to that of Fe^3+^ ^21–23^. Consequently, Zn^2+^ was used as an alternative to Fe^3+^ in soaking experiments with ascorbate.

**Fig. 2:**
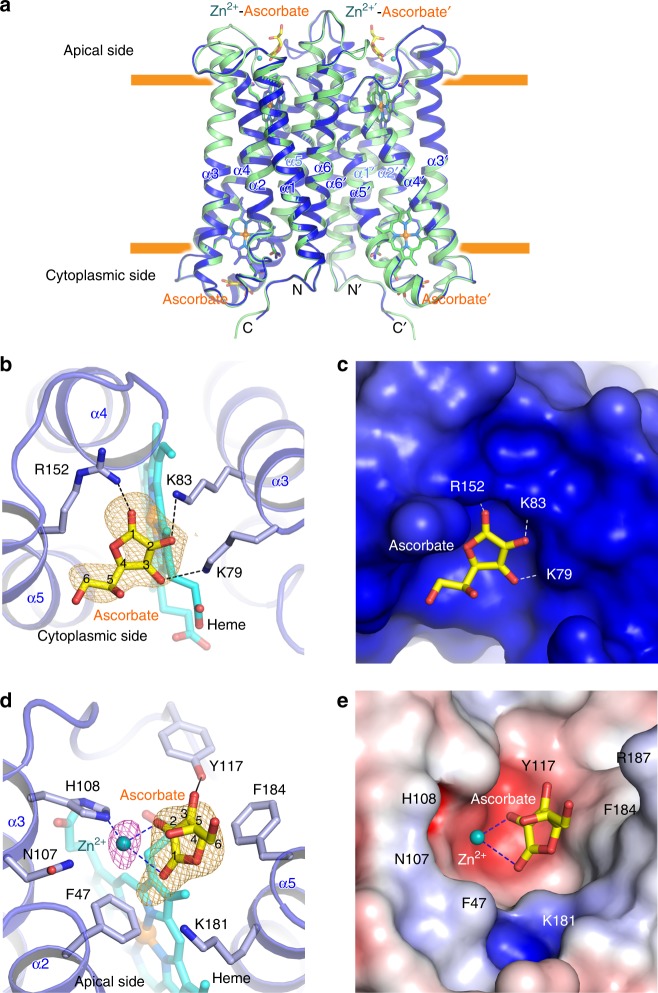
Substrate-bound structure of Dcytb. **a** The six α-helices of the substrate-free (green) were superimposed on those of the substrate-bound structure (blue) with an r.m.s.d. of 0.4 Å for all Cα atoms. **b** Ascorbate is bound on the cytoplasmic surface of Dcytb by interacting with three positively charged residues. The omit map for ascorbate is contoured at 2.5σ (orange mesh). **c** The negatively charged cavity for ascorbate-binding on the cytoplasmic surface is shown. **d** The Zn^2+^ binding to H108 and two hydroxyl groups of ascorbate on the apical surface of Dcytb is shown. The anomalous difference Fourier map (magenta mesh) calculated from X-ray data collected at 1.0 Å is contoured at 3.5σ. The omit map for ascorbate (orange mesh) bound to the apical side is contoured at 4.0σ. **e** The Zn^2+^-ascorbate-binding cavity on the apical surface is shown

Overall, the structure of Dcytb with Zn^2+^ and ascorbate bound as determined at 2.8 Å resolution exhibited no significant conformational change from the structure of Dcytb determined in the absence of Zn^2+^ and ascorbate (r.m.s.d. 0.4 Å for all Cα atoms) (Fig. 2a) except for the disordered electron density of F47 (Supplementary Fig. 3). Clear electron density for substrate binding was observed at both the cytoplasmic and apical sides of the protein. The electron density from one ascorbate molecule was observed on the cytoplasmic surface of Dcytb in proximity to the heme located near the cytoplasmic surface (Fig. 2b). Note that ascorbate cannot be distinguished from the oxidized form, monodehydroascorbate, in these results, so for simplicity we refer only to ascorbate in this report. Consistent with the properties of ascorbate, the ascorbate-binding pocket contains three positively charged residues: the 1-ketone group of ascorbate is hydrogen bonded with R152 from α5, and the hydroxyl groups at positions −2 and −3 are hydrogen bonded with K83 and K79 from α3, respectively (Fig. 2b, c). These three residues are highly conserved in the Cytb_561_ family. In addition, the 5-methyl group of the cytoplasmic heme group is within van der Waals distance of the lactone ring of ascorbate. The importance of these three residues in the ascorbate-binding site was investigated by stopped-flow kinetics studies of ascorbate reduction of the K79S, K83S and R152E variants (Supplementary Fig. 4 and Supplementary Table 1). The rate of heme reduction was decreased by these substitutions. Similar results were reported following substitution of the corresponding residues in *At*Cytb_561_^20^. These kinetic results provide evidence that these three residues contribute to efficient electron transfer from the cytoplasmic ascorbate to the adjacent heme group of Dcytb. The protein−ascorbate interaction observed in cytoplasmic surface of Dcytb shows some similarity to that in ascorbate peroxidase (APX)^24^ in which two negatively charged residues (K30 and R172) interact with ascorbate. However, these two sites differ in that a heme propionate and main chain carbonyl group also interact directly with ascorbate in APX and that the location and orientation of ascorbate with respect to the heme differ.

The metal ion binding site was identified by an anomalous difference Fourier map (Fig. 2d). Specifically, a strong electron density observed on the apical side of Dcytb near the apical heme and H108 was assigned to Zn^2+^. This Zn^2+^ is coordinated to the Nε_2_ atom of H108 at a distance of 2.0 Å. In addition to the density for Zn^2+^, an additional density was observed very close to the Zn^2+^. This extra density appears as a flat shape in the *F*_o_ − *F*_c_ map that occurs parallel to F184 and that we attribute to a bound ascorbate (Fig. 2d). This arrangement means that Zn^2+^ binding to Dcytb involves coordination to ascorbate. The distances of Zn^2+^ to the O-2 and O-1 atoms of ascorbate are 2.2 Å and 2.8 Å, respectively, and the Nε_2_(H108)-Zn^2+^-O-2(ascorbate) angle is 89°. N107 is not coordinated to Zn^2+^ but is nearby (3.4 Å). The only other interaction of ascorbate with the protein occurs through van der Waals contact with F184, which resides 3.5–4.0 Å from the lactone ring of ascorbate. The weak electron density of the ascorbate dihydroxyethyl group (C5 and C6 position) probably results from lack of any interaction with the protein. Although the side chains of Y117, K181 and R187 contribute to the formation of the large pocket on the apical surface, these residues do not interact directly with ascorbate through hydrogen-bonding or salt bridge formation (Fig. 2d, e).

### Analysis of metal binding by resonance Raman spectroscopy

**Fig. 3:**
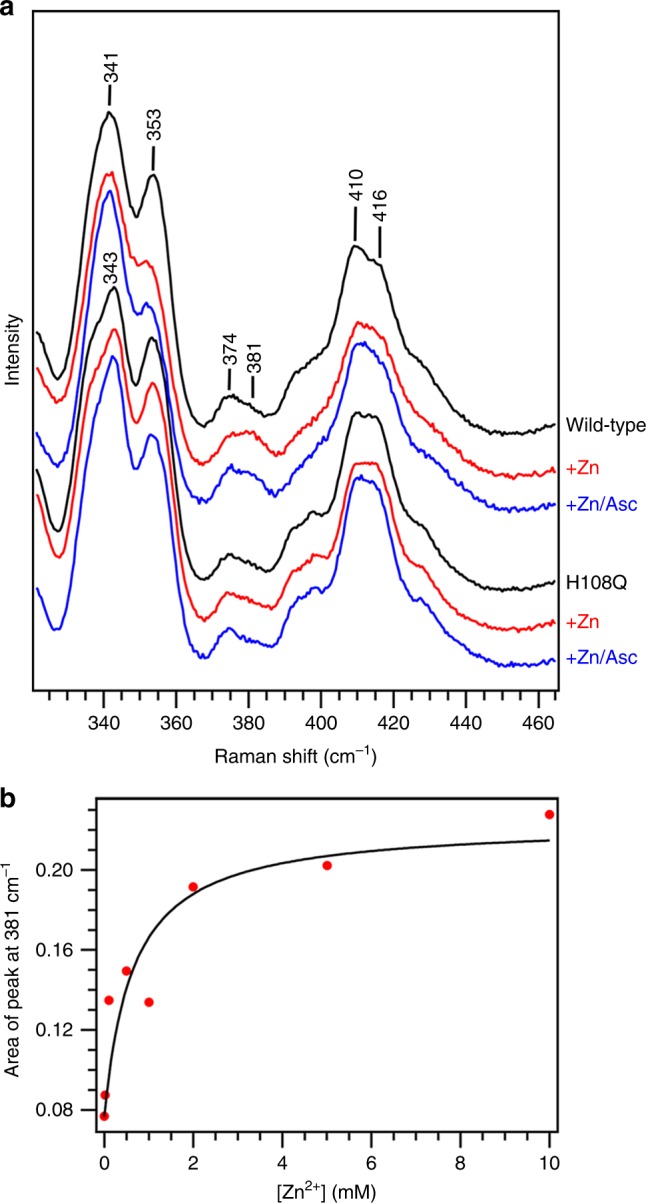
Resonance Raman analysis of Zn^2+^ binding to reduced Dcytb. **a** The low frequency region of the resonance Raman spectrum of Dcytb in the absence and presence of ZnCl_2_ (5 mM) or ZnCl_2_ (5 mM) and ascorbate (20 mM) are shown for the wild-type and H108Q variant proteins. **b** The titration of reduced wild-type Dcytb with Zn^2+^ as monitored by resonance Raman spectroscopy at 381 cm^−1^. The peak areas at 381 cm^−1^ are plotted as red circles as a function of [Zn^2+^]. The black curve represents a fit of the data to Eq. 2 to provide an estimated *K*_d_ of 0.5 ± 0.1 mM

To characterize Zn^2+^-binding to H108 of Dcytb further, we studied wild-type Dcytb and the H108Q variant in the presence and absence of ascorbate and Zn^2+^ by resonance Raman spectroscopy (Fig. 3). As the heme irons of Dcytb are partially reduced upon laser irradiation, we focused on Zn^2+^ binding to the reduced form, but not the oxidized form, of Dcytb. We collected spectra of Dcytb in the presence of Na_2_S_2_O_4_ to assure that the heme irons were fully reduced. Upon addition of ascorbate to either protein, no spectroscopic changes were observed in either the high or low frequency regions, indicating that ascorbate binding to Dcytb induces no significant structural changes around the hemes. On the other hand, addition of Zn^2+^ to wild-type Dcytb in the presence or absence of ascorbate induced the appearance of a small band at 381 cm^−1^ in the low frequency region. Since the 381 cm^−1^ band was assigned as a bending vibration of the heme propionate groups^25^, the Zn^2+^ binding could induce the change of the environment of the heme propionate group. This change in spectrum was not observed following addition of Zn^2+^ or a mixture of Zn^2+^ and ascorbate to the H108Q variant. H108 is located adjacent to a propionate group of the apical heme, so the appearance of the 381 cm^−1^ band in the presence of Zn^2+^ is consistent with participation of H108 in the binding of this metal ion as indicated by the crystallographic structure. By monitoring the increase in intensity of the 381 cm^−1^ band while titrating Dcytb with Zn^2+^, the dissociation constant for Zn^2+^ binding was estimated to be ~0.5 mM (Fig. 3b and Supplementary Fig. 5).

We also examined the Fe^3+^ and Cu^2+^ titration of fully reduced Dcytb in the presence of Na_2_S_2_O_4_ but did not observe any change in the low frequency region of the resonance Raman spectrum. As observed during crystallization experiments, Fe^3+^ and Cu^2+^ can be reduced readily to Fe^2+^ and Cu^1+^, neither of which binds to Dcytb as observed by resonance Raman spectroscopy. This observation is consistent with the physiological expectation that both Fe^2+^ and Cu^1+^ should dissociate from Dcytb for subsequent transport by DMT-1.

### Analysis of electron transfer pathway in Dcytb

**Fig. 4:**
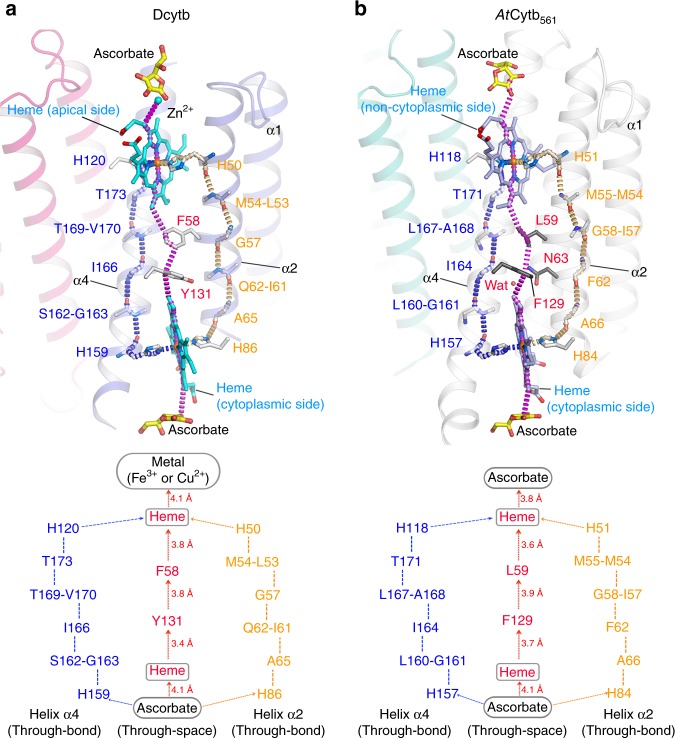
Proposed electron transfer routes of Dcytb and *At*Cytb_561_. **a** In Dcytb, three possible routes can be proposed based on the current structure: First, through-space electron transfer (magenta dashed line) mediated by two aromatic residues (Y131 and F58), which are aligned within the distance of 4 Å. The F58 is substituted with Leu, Tyr or Met in most of other members of Cytb_561_ family, whereas Y131 is conserved or substituted with Phe in other members. Second, through-bond electron transfer (orange dashed line) mediated by a series of amino acid residues along α-helix 2. Third, through-bond electron transfer (blue dashed line) mediated by a series of amino acid residues along α-helix 4. **b** In *At*Cytb_561_, an F129- and L59-mediated route (magenta dashed line), a route involving covalent and H-bonds along α-helix 2 (orange dashed line), and a route along α-helix 4 (blue dashed line) were suggested. A water-mediated route (through space) has also been proposed for *At*Cytb_561_ by Lu et al.^20^, although we could not reproduce it. The binding of water to N63 of *At*Cytb_561_ might be not conserved in Cytb_561_ family because N63 is diverse. Indeed, this position corresponds to Q62 in Dcytb, a substitution that results in no space for water

The possible pathways for electron transfer from the cytoplasmic ascorbate to iron bound at the apical surface were explored with the *Pathways* plugin for VMD^26,27^. These calculations suggest three possible electron transfer pathways as shown in Fig. 4: first, [cytoplasmic heme]→Y131→ F58 → [apical heme] → Fe^3+^ (or Cu^2+^). This pathway includes three through-space transfers between the two hemes that have distances of 3.6~4.9 Å; second, a pathway through a series of covalent and H-bonds along the helix α2 (orange dashed line); and third, a pathway along the helix α4 (blue dashed line). These results are compared with those obtained from identical calculations for *At*Cytb_561_ in Fig. 4. Despite the differences in structure exhibited by these two proteins in the region between the two heme groups of each monomer, these calculations indicate that the pathways for electron transfer between two heme groups of each of these two proteins are remarkably similar.

### Ferric reductase activity of Dcytb

As shown above (Fig. 2d), H108, Y117, F184, and N107 contribute to the environment in the region of metal ion and ascorbate binding. To evaluate the functional roles of these residues, site-directed mutants of human Dcytb were expressed in a yeast strain (*Saccharomyces cerevisiae fre1*Δ*fre2*Δ) that lacks the endogenous plasma membrane Fe^3+^ reductases, Fre1 and Fre2. Expression of wild-type human Dcytb on the plasma membrane of this yeast mutant rescues the growth of this yeast by providing Fe^3+^ reduction required for nutritional iron absorption that is normally provided by Fre1 and Fre2. In this system, electrons for Fe^3+^ reduction are supplied by cytoplasmic ascorbate produced by yeast ascorbate biosynthesis^28^.

**Fig. 5:**
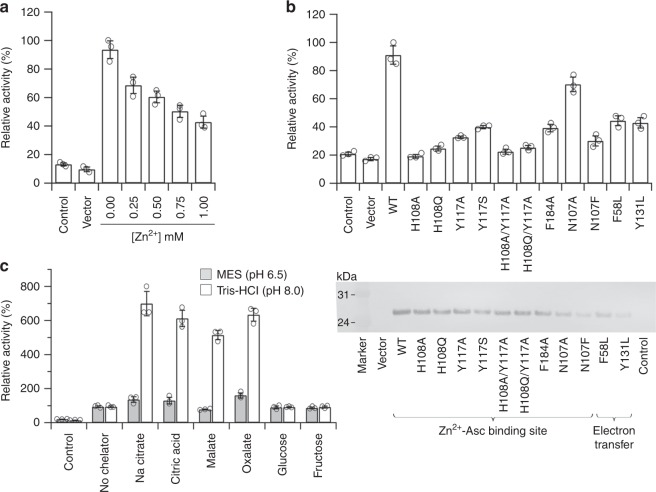
Ferric reductase activity of Dcytb. The values indicated are means ± S.D. of data from duplicate sets of samples analyzed in triplicate. The “Control” means that the Dcytb expression was suppressed in the transformant of wild-type *dcytb* cDNA-inserted vector, and the “Vector” shows that the expression vector (no insertion of *dcytb* gene) was transformed and the assay was performed in the same manner as with cultures of the wild-type and other variants. Dot-plot (empty circles) shows the data distribution. **a** Competitive binding between Fe^3+^ and Zn^2+^ in wild-type Dcytb. The value of 0 mM (~220 nmol 10^6^ cells^−1^ h^−1^) was set to 100% and the relative activity was obtained for increasing Zn^2+^ concentration. **b** Fe^3+^ reductase activities of the structure-guided mutants of Dcytb. Western blot of whole cell lysates of Dcytb-expressed yeasts (1.0 μg protein per lane) shown at the bottom. The original blot is represented in Supplementary Fig. 7. **c** Effect of dietary metal-chelators on Fe^3+^ reductase activity in wild-type Dcytb. The effect of chelators was tested in MES buffer (pH 6.5) and Tris-HCl buffer (pH 8.0). The activity without chelator for MES buffer (~195 nmol 10^6^ cells^−1^ h^−1^) and Tris-HCl buffer (~65 nmol 10^6^ cells^−1^ h^−1^) was set to 100%, and the relative activity with chelators was calculated for respective buffers

With this growth assay, we first examined the effect of Zn^2+^ binding on the Fe^3+^ reductase activity of wild-type and variant forms of Dcytb (pH 6.5, sodium citrate buffer (50 mM), 5% w/v glycerol, conditions similar to those in the duodenum^29^). As [Zn^2+^] was increased to 1.0 mM, ferric reductase activity of wild-type Dcytb decreased by about 43% (Fig. 5a), consistent with Zn^2+^ binding to the enzyme competitively with Fe^3+^.

The functional roles of specific residues were studied next by evaluating the activities of several Dcytb variants (Fig. 5b). The H108A and H108Q variants exhibited the lowest Fe^3+^ reductase activities (20 and 25%, respectively) of those studied. These results are consistent with the proposal that H108 is a ligand for the binding of Fe^3+^ as well as Zn^2+^. Although N107 is not a Zn^2+^ ligand, the amide nitrogen is just 3.7 Å from the bound metal ion, so presumably the site for binding of the Fe^3+^-ascorbate complex in the structure of the N107F variant is perturbed sufficiently to account for the 35% activity resulting from this substitution. Interestingly, even though Y117 is ~6.6 Å from the bound Zn^2+^, the Y117A and Y117S variants exhibited ~35 and 40% of the wild-type activity. Considering the relative proximity of Y117 to the adjacent ascorbate, it seems likely that the decreased activities of these variants result from perturbation of ascorbate binding, which would, in turn, affect binding of Fe^3+^. A similar mechanism presumably accounts for the decreased activity exhibited by the F184A variant (~35% of wild-type activity). Finally, the proposal that F58 and Y131 participate in the electron transfer pathway between the cytoplasmic and apical heme groups is consistent with the observation that the activities of the F58L and Y131L variants were less than 50% of wild-type Dcytb.

Ferric iron is presented to Dcytb in the duodenum in a variety of chelated forms because many potential soluble ligands for iron occur in this environment and in nutritional sources of iron. The relatively accessible binding sites for iron and ascorbate on the apical surface of Dcytb appear to be compatible with this multiplicity of substrate forms (Fig. 2d), but the influence of likely iron ligands on Dcytb activity is unknown. To investigate this issue, the effect of soluble chelators on the Fe^3+^ reductase activity of Dcytb was evaluated by the individual supplementation of the yeast assay mixture with a variety of putative dietary chelating agents (Fig. 5c). Citrate, which was used as a buffer in the kinetics experiments above, is a known dietary chelator that supports iron uptake from the gut^30^. As an excess of citrate may reasonably be expected to occupy the relatively accessible binding site for Fe^3+^ and ascorbate, the effect of soluble chelators on Fe^3+^ reduction was evaluated in nonchelating buffers. In addition, because the pH of digesting nutrients changes from acidic to basic with passage from the stomach to the duodenum^29^, we studied Fe^3+^ reduction by Dcytb under acidic (MES, pH 6.5) and basic (TrisHCl, pH 8.0) conditions (Fig. 5c). These studies revealed that at acidic pH, the effect of added ligands on reductase activity was oxalate > citrate > no chelator > glucose~fructose > malate while at basic pH, this order was changed to citrate~oxalate > malate > no chelator~glucose~fructose. Notably, the ability of citrate, malate, and oxalate to accelerate Fe^3+^ reduction by Dcytb is far greater at alkaline pH than at acidic pH.

## Discussion

Human ferric reductase Dcytb is an essential enzyme for enteric iron absorption and a member of Cytb_561_ protein family, which has two heme b groups to mediate intramolecular electron-transfer across the membrane. The principal substrate of the enteric enzyme Dcytb is Fe^3+^ while that of the adrenal and plant enzymes is monodehydroascorbate. The adrenal enzyme resides in chromaffin granules (CGcytb) and reduces intravesicular monodehydroascorbate for regeneration of ascorbate that is used in the biosynthesis of the neurotransmitter norepinephrine^31^. Nevertheless, the CGcytb and the lysosomal enzyme (Lcytb) have also been shown to be capable of catalyzing Fe^3+^ reduction by ascorbate^32^.

The sequence of the human Dcytb core structure (residues 6–230) shares 36% identity and 52% similarity with that of the plant enzyme *At*Cytb_561_ (residues 9−220), the only other member of this family for which a crystallographically determined structure has been reported^20^. Comparison of these two structures reveals that the six α helices (163 Cα atom pairs) are superimposable with an r.m.s.d. of 0.6 Å despite the conformational differences observed in the loop regions that connect the α helices. Moreover, the relative orientations of monomers within these two dimers differ by only 5°. The overall structure is well conserved among all Cytb_561_ family members.

One significant difference in the sequence of Dcytb relative to other members of this family is the presence of an additional 56-residue C-terminal region that is absent from the *At*Cytb_561_ MDA reductase. This region is located on the cytoplasmic side of the protein and is disordered in the structure of Dcytb reported here. Three human proteins belonging to the Cytb_561_ family, Dcytb (duodenal brush border membrane), Lcytb (late endosomal-lysosomal membrane), and CGcytb (chromaffin granule), differ in the length of this C-terminal region (Supplementary Fig. 6) and in their cellular locations. Considering that Dcytb and Lcytb act as Fe^3+^ reductases while CGcytb acts as an MDA reductase, this C-terminal region may be related to the subcellular locations of the enzymes or to their physiological functions. Indeed, the C-terminal region of human Lcytb was found to contain the signal sequence that targets this enzyme to the lysosomal membrane^33^. One example of a physiological role for such sequences in another transmembrane protein involved in iron metabolism includes the regulatory role demonstrated for one of the cytoplasmic loops of DMT-1 in interaction with an iron chaperone protein^34^. Similarly, a cytoplasmic lysyl residue of the iron exporter ferroportin-1 is a ubiquitination site^35^. Based on these precedents, it seems likely that the disordered C-terminal region of Dcytb may have a role in the regulation of duodenal iron absorption. Clearly, further studies are required to evaluate the functional role of the C-terminal region of Dcytb.

Comparison of the structures of Dcytb and *At*Cytb_561_ also reveals that the electrostatic potential of the apical surface of Dcytb is far more negatively charged (acidic) than that of the corresponding region of the plant enzyme. This characteristic contrasts with the corresponding surfaces of *At*Cytb_561_, both of which are basic but not as basic as the cytoplasmic surface of Dcytb. The negatively charged apical surface of Dcytb is attributable to the presence of D188 (N186), E36 (G35), D41 (D42), E106 (K104), and E197 (E195) (the corresponding residues of the *At*Cytb_561_ enzyme are indicated in parentheses). This surface may assist in directing Fe^3+^ complexes toward the active site of the reductase to promote reaction.

The structural environments of the two heme groups of Dcytb are nearly identical to those of the heme groups occurring in *At*Cytb_561_ in terms of bis-histidyl coordination and interactions with surrounding residues. On the other hand, the structure of the protein intervening between the two heme groups that is expected to participate in intramolecular electron transfer differs significantly between Dcytb and *At*Cytb_561_. For both proteins, the shortest distance between the two heme centers is 15.5 Å. Inspection of the *At*Cytb_561_ structure led Lu and co-workers to propose involvement of a water molecule and F129 (located 3.5 and 3.6 Å from the cytoplasmic heme, respectively). Lu et al. suggested that the configuration of this water and the phenyl ring of Phe129 is suitable for electron transfer tunneling from the cytoplasmic heme to the noncytoplasmic heme^20^. In addition to this through-space route, a through-bond route involving residues of transmembrane helix-2 was also suggested for *At*Cytb_561_ from computational analysis. However, no experimental investigation of the mechanism of electron transfer between the heme groups of *At*Cytb_561_ has been undertaken^20^. In the case of Dcytb, a putative through-bond electron transfer routes can be identified through main chain NH–OC interactions from the cytoplasmic heme ligand through six residues along helix α2 or α4, and, ultimately, the apical heme ligand (Fig. 4). Notably, the putative through-space pathway differs between Dcytb and *At*Cytb_561_ in that (a) F129 of *At*Cytb_561_ corresponds to Y131 in Dcytb, (b) no water molecule occurs in this pathway for Dcytb, and (c) the phenyl group of Dcytb residue F58 resides between Y131 and the heme while L59 occupies this position in *At*Cytb_561_ (Fig. 4). Consequently, Dcytb residues F58 and Y131 could promote electron tunneling over a reasonable distance (3.6−4.9 Å) (Fig. 4). Such a proposal is consistent with the in vivo Fe^3+^ reductase assay analysis of intramolecular electron transfer route of Dcytb variants with substitutions at these positions (Fig. 5b).

**Fig. 6:**
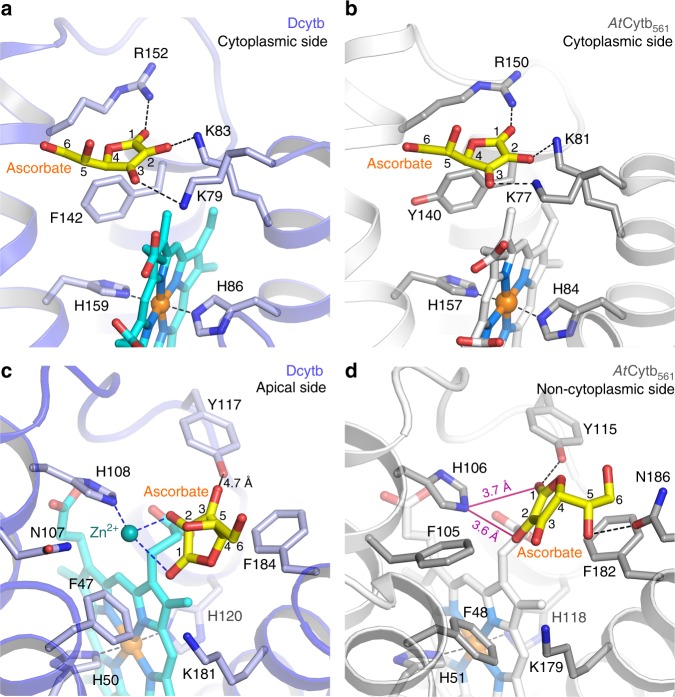
Comparison of the cytoplasmic and apical substrate-binding sites of Dcytb and *At*Cytb_561_. **a** Ascorbate is bound to the cytoplasmic side of Dcytb by interaction with three conserved residues (K79, K83, and R152). F142 interacts with ascorbate by van der Waals contact. **b** Similarly, ascorbate is bound to the cytoplasmic side of *At*Cytb_561_ by three conserved cationic residues, and Y140 replaces F142 of Dcytb. **c** Zn^2+^-ascorbate is bound to the pocket on the apical side of Dcytb. Zn^2+^ is coordinated by H108 and two-hydroxyl groups of ascorbate. H108 is conserved in all members of Cytb_561_. Ascorbate is in van der Waals contact with F184. The dihydroxyethyl moiety of ascorbate is free from interaction with the protein. **d** Ascorbate is bound to the same site on the noncytoplasmic side of *At*Cytb_561_. Ascorbate is in van der Waals contact with H106 and F182 and forms H-bonds with Y115 and N186

The binding sites for ascorbate on the cytoplasmic sides of Dcytb and *At*Cytb_561_ are identical and are defined by the conserved residues K79 (77), K83 (81), and R152 (150) (Fig. 6a, b). This structural and functional conservation is not surprising as all members of the Cytb_561_ family employ ascorbate as a cytoplasmic electron donor. On the other hand, the ascorbate-binding sites at the apical surface of the protein differ between Dcytb and *At*Cytb_561_ in a manner that presumably reflects the differing structural requirements for Fe^3+^ reduction and monodehydroascorbate reduction. The identification of the Zn^2+^ binding site on Dcytb that is located adjacent to the apical binding site for ascorbate provides structural evidence for Cytb_561_ involvement in metal ion reduction. The cooperative binding of ascorbate and Zn^2+^ to the apical substrate binding site in the Dcytb structure reveals key mechanistic insight into Dcytb function. Other Cytb_561_ family members are capable of reducing monodehydroascorbate to ascorbate at the expense of ascorbate, which binds to the cytoplasmic substrate binding site, to provide electron to monodehydroascorbate on the apical side. Dcytb possesses an ascorbate-binding site at the cytoplasmic surface of the protein as do other members of the Cytb_561_ family, but it also possesses an apical binding site for ascorbate that integrates a binding site for the Fe^3+^ substrate that could lead to formation of monodehydroascorbate upon reduction of Fe^3+^ to Fe^2+^. The resulting monodehydroascorbate could then be reduced back to ascorbate by Dcytb. This mechanism suggests that Dcytb not only reduces Fe^3+^ but also regenerates ascorbate from monodehydroascorbate on the apical binding site using electrons provided by ascorbate at the cytoplasmic binding site.

**Fig. 7:**
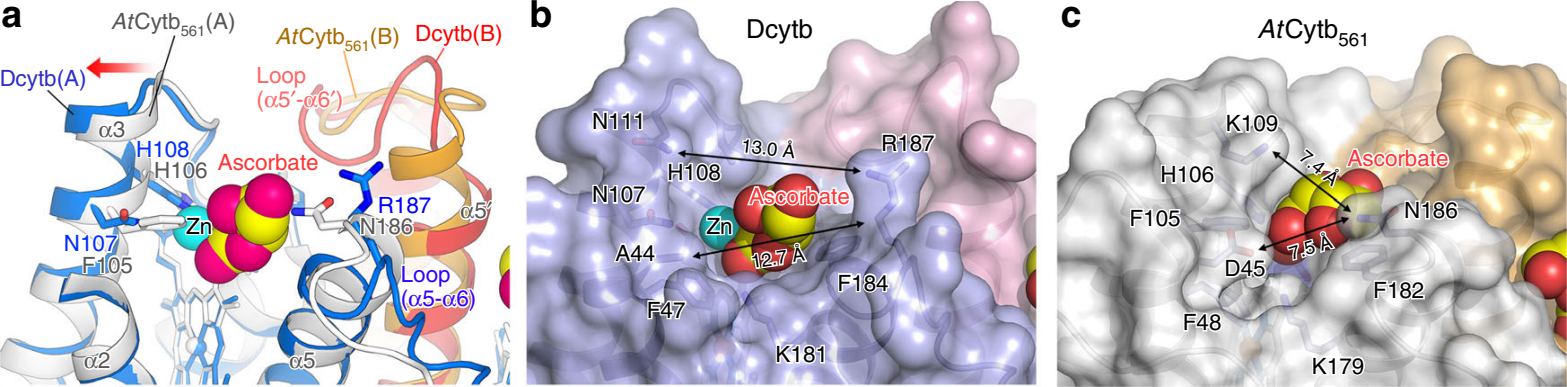
Comparison of the binding pockets on the apical surfaces of Dcytb and *At*Cytb_561_. **a** Superposition of Dcytb and *At*Cytb_561_. The α3 helix is bent in the middle to accommodate binding of Zn^2+^ and ascorbate. The loop connecting α5–α6 contributes to this binding pocket and exhibits significant conformational difference between the two proteins. Dcytb (A-chain (blue), B-chain (red)) and *At*Cytb_561_ (A-chain (gray), B-chain (orange)) are colored as indicated. The A-chains of the two proteins were superimposed for calculations. Space filling models are shown for Zn^2+^ and ascorbate bound to Dcytb. The α2 helices of the A-chains of both proteins are excluded from this view for clarity. **b** The binding pocket of Dcytb is wide and open above the ligand. This view is the same as shown in **a**. **c** Ascorbate in the binding pocket of *At*Cytb_561_ is more buried

H108, Y117, and N107 are all identified by the structure of Dcytb as participating directly or indirectly in the binding of Zn^2+^ and, by implication, in the binding of Fe^3+^. H108 plays a functionally conserved role in ascorbate binding at the apical surface of both Dcytb and *At*Cytb_561_ and, in providing a ligand for Zn^2+^ binding, plays a direct role in the binding of this metal ion. Y117 is too distant (6.6 Å) from Zn^2+^ to serve as a ligand, and it is just beyond hydrogen-bonding distance from two ascorbate hydroxyl groups (4.7 Å). But the high affinity of a tyrosine phenolate for Fe^3+^ and the possible contribution of this residue to optimal orientation of bound ascorbate raise the possibility that Y117 might influence the Fe^3+^ reductase activity of this enzyme. Finally, the replacement of F105 in *At*Cytb_561_ by N107 in Dcytb appears to create an open space for metal ion binding (Fig. 6c, d). Superposition of the Dcytb and *At*Cytb_561_ structures (Fig. 7) suggests that the loop region between α5 and α6 also contributes to creation of this open space, which is of sufficient size to accommodate metal-chelator complex binding as discussed below.

Another significant finding in the present study is that ascorbate, citrate, and some organic acids can assist metal-binding to the apical side of Dcytb as Fe^3+^-chelators. The solubility and oxidization state of nonheme iron are highly affected by physiological pH. Both Fe^3+^ and Fe^2+^ are soluble at the low pH present in the stomach, but as pH increases upon passage into the duodenum, precipitates form unless iron is bound by soluble iron chelators^36^. Our functional assay with some dietary metal-chelators revealed that citric acid, malic acid, and oxalic acid promote Fe^3+^ reduction by Dcytb at duodenal pH. These results support the conclusion that the Fe^3+^ binding site of Dcytb is readily accessible to interaction with Fe^3+^-chelator complexes. Notably, although oxalate is a known inhibitor of iron uptake^37^, it enhanced Fe^3+^ reduction by Dcytb at both acidic and basic pH, so the inhibitory effect of oxalate on iron absorption is not the result of its effect on Fe^3+^ reduction. Interestingly, sugars such as glucose and fructose, which are found in oral iron supplements, have little or no effect on Dcytb activity even though they are known to potentiate iron uptake^38,39^. Understanding of such interactions could be useful in the development of new and effective therapeutic agents to promote iron uptake.

In summary, iron deficiency continues to be a significant cause of malnutrition in both developing and industrialized nations, in part because intestinal absorption of dietary iron is relatively inefficient. The current study provides structural insight into the function of one of the two duodenal proteins that are required for the uptake of dietary nonheme iron. In combination with the current biochemical studies, this work also provides structural insight into how ascorbate enhances iron uptake and how several potential iron ligands found in various foods promote or inhibit iron uptake. Ultimately, knowledge of the structure of Dcytb should enable development of new, structure-based strategies for promoting the reduction of dietary iron and thereby enhancing the bioavailability of this essential nutrient.

## Methods

### Expression and purification of Dcytb

A codon-optimized gene encoding Dcytb that includes a thrombin cleavage site followed by a C-terminal hexa-His tag was cloned into the pET-21b(+) vector (Merck Millipore) for expression in *Escherichia coli* BL21 Star(DE3) (Invitrogen) and grown in TB medium containing δ-aminolevulinic acid (ALA) (0.5 mM) and ampicillin (50 mg L^−1^). Overexpression of Dcytb was induced by isopropyl β-d-thiogalactoside (0.1 mM) when the culture achieved an OD_600_ of 1.5–1.7, and cell growth was continued for 18 h at 20 °C. The harvested cells were suspended in phosphate-buffered saline (pH 7.0) containing DNase I (10 mg mL^−1^), lysozyme (20 mg mL^−1^) and one tablet of cOmplete EDTA-free protease inhibitor cocktail (Roche) followed by disruption with a French press (Ohtake). Cell debris was removed by centrifugation (7400 × *g* for 30 min with an R13A angle rotor and CR22N centrifuge (Hitachi)). The supernatant fluid was collected and sedimented by ultracentrifugation (66,000 × *g* for 1 h with a P45AT angle rotor and CP80WX ultracentrifuge (Hitachi)) to obtain the membrane fraction. This fraction was then homogenized with lysis buffer (Na_2_HPO_4_ (50 mM), citric acid (10 mM) and NaCl (150 mM), pH 5.8) and solubilized in a solution containing sodium deoxycholate (0.5% (w/v)) (Wako Pure Chemical) and n-dodecyl-β-d-maltopyranoside (DDM) (1.7% (w/v)) for 1 h at 4 °C. Following ultracentrifugation (40,000 rpm for 1 h), the supernatant fluid was loaded onto a column of Ni^2+^-nitrilotriacetate affinity resin (Ni-NTA; Qiagen). Nonspecifically bound material was eluted from this column with lysis buffer containing 10 mM imidazole (pH 5.8) (washing buffer), and the solubilized Dcytb was eluted by the same buffer containing 500 mM imidazole (pH 5.8). The hexa-His tag was cleaved by overnight treatment with biotinylated thrombin (Merck Millipore) at 20 °C followed by incubation of this reaction mixture with streptavidin agarose (Merck Millipore) for 1 h at 4 °C to capture the biotinylated thrombin. The resulting protein solution was loaded into a column of Ni-NTA, and the His-tag-cleaved Dcytb was eluted with washing buffer. The resulting Dcytb solution was concentrated to ~4–5 mg mL^−1^ by centrifugal ultrafiltration and purified by gel filtration chromatography (HiLoad 16/600 Superdex 200 pg; GE Healthcare) equilibrated with MES-Na buffer (50 mM, pH 6.5) containing NaCl (150 mM) and DDM (0.025%). The peak fractions were collected and concentrated (~15 mg mL^−1^) for crystallization with a centrifugal ultrafiltration unit (Amicon, Merck Millipore) having a 50 kDa cutoff.

### Crystallization

Purified Dcytb was crystallized by the LCP method. Protein solution (~15 mg mL^−1^) was mixed with monoolein (9.9 MAG; Nu-Chek Prep, Inc) at a 2:3 ratio (w/w) with a coupled syringe mixer. Samples (50 nL) of protein-laden LCP were dispensed onto a 96-well sandwich plate (Molecular Dimensions) and overlaid with precipitant solutions (0.8 μL) with a mosquito LCP device (TTP Labtech). Initial crystallization hits were identified in the presence of 20% (v/v) Jeffamine ED-2003 and 0.1 M HEPES (pH 6.5). Crystallization conditions were optimized by screening further with buffer containing a series of Jeffamine concentrations and varying pH. Crystals that reached full size (~4–8 μm) within 2 days at 20 °C were harvested from the mesophase and were flash cooled in liquid nitrogen without additional cryoprotectant. Crystals of Dcytb with substrate bound were obtained by soaking crystals grown for 2 weeks at 20 °C with precipitant solution containing sodium ascorbate (1 M) and ZnSO_4_ (10 mM) for 30 min and, finally, flash cooled in liquid nitrogen. The reasons why we used high concentration of ascorbate in the soaking experiment are: first, ascorbate affinity of Dcytb is not high. When we used lower concentration of ascorbate for the crystal soaking, the electron density of ascorbate was very weak; second, Dcytb crystals were prepared by LCP method and sandwiched by two glass plates. The diffusion rate of ascorbate into the crystals in the lipid was slow and therefore the soaking experiment was technically not easy, and third, it was reported that the soaking condition of *At*Cytb_561_ crystals was 1 M ascorbate^20^.

### Data collection and refinement

X-ray diffraction data were collected at a wavelength of 1.0 Å (100 K) on beamline BL32XU at SPring-8 with an EIGER X 9 M (Dectris) detector. The ZOO and KAMO^40^ (https://github.com/keitaroyam/yamtbx) systems were used for the automated crystal mounting, loop centering, data collection and data processing. From hundreds of crystals, small-wedge data (5° per crystal) were collected using 5 × 5 μm^2^ beam. All datasets were indexed and integrated by XDS^41^ and those indexed with similar latice parameters were scaled and merged with outlier rejections implemented in KAMO. For Zn^2+^-ascorbate-bound form, hierarchical clustering analysis based on unit cell parameters were performed using BLEND^42^ prior to merging. The best cluster was selected based on the anomalous difference Fourier peak height at Zn^2+^. Data collection statistics are summarized in Table 1. An initial model of Dcytb was obtained by molecular replacement with Phaser^43^. *Arabidopsis thaliana* Cytb_561_ (PDB code: 4O6Y) was used as the search model. The structure was refined with PHENIX^44^, and model building was carried out with Coot^45^. The Ramachandran plot for refined models gave 99.6% of nonglycine residues in favored region and 0.4% of residues in the disallowed region. ANODE^46^ was used to calculate an anomalous difference Fourier map. The refinement statistics are summarized in Table 1. All structural figures were prepared with the PyMOL Molecular Graphics System (Version 2.0 Schrödinger, LLC).

**Table 1:** Statistics of X-ray diffraction data collection and refinement

	Dcytb (substrate-free)	Dcytb (Zn^2+^ and Asc bound)
Data collection		
Beamline (SPring-8)	BL32XU	BL32XU
Space group	*C*2	*C*2
Wavelength (Å)	1.0	1.0
Cell dimensions		
* a*, *b*, *c* (Å), *β* (°)	64.45, 115.94, 48.43, 118.45	65.19, 115.57, 48.60, 118.44
Resolution (Å) ^*a*^	50−2.6 (2.76–2.60)	50–2.8 (2.97–2.80)
Nr of merged crystals	866	600
Observed reflections	698,003	387,956
Unique reflections	9644	7824
* R*_pim_ (%)^a,b^	11.8 (91.2)	22.5 (306.9)
Average *I/*σ(*I*)^a^	10.0 (1.1)	8.5 (1.0)
* CC*_1/2_ (%)	98.6 (68.8)	96.7 (48.1)
Completeness (%)^a^	99.3 (99.3)	99.3 (99.7)
Redundancy^a^	72.4 (68.7)	49.6 (48.8)
Wilson *B*-factor (Å^2^)	26.9	30.9
Refinement		
* R*_work_/*R*_free_ (%)^*c*^	19.7/24.5	20.6/25.3
No. atoms		
Protein	1797	1797
Heme	86	86
Zn^2+^-ascorbate	0	25
Water	5	3
Average *B* factors		
Protein	34.0	35.4
Heme	24.0	24.1
Zn^2+^-ascorbate	—	54.5
Water	29.3	18.2
R.m.s.d. bond (Å)	0.008	0.009
R.m.s.d. angles (°)	1.19	1.25
PDB entry	5ZLE	5ZLG
SBGrid entry	573	574

^a^Values in parentheses are for the highest-resolution shell

^b^*R*_pim_ = Σ_*hkl*_{1/(*n* − 1)}^1/2^ Σ_*i*_|*I*_*i*_(*hkl*)−〈*I*(*hkl*)〉|/Σ_*hkl*_Σ_*i*_*I*_*i*_(*hkl*), where *n* is the multiplicity of reflection *hkl*, and 〈*I*(*hkl*)〉 is the average intensity of *i* observations

^*c*^ *R*_work_ = Σ_*hkl*_|*F*_obs_(*hkl*) − *F*_calc_(*hkl*)|/Σ_*hkl*_*F*_obs_(*hkl*), where *F*_obs_ and *F*_calc_ are the observed and calculated structure factors, respectively. *R*_free_ was calculated with 5% of the reflections

### Stopped-flow measurements

The rates of the reduction of wild type and variants of Dcytb by ascorbate were determined by monitoring the absorbance change at 427 nm with a stopped-flow spectrophotometer (UNISOKU RSP-1000). For most experiments, concentrations of Dcytb in the syringe of the stopped-flow instrument were 2 μM in the sample buffer containing HEPES (25 mM, pH 7.0, with NaCl (150 mM) and DDM (0.05% (w/v))). Protein solutions were degassed and placed under nitrogen prior to loading into the syringes. For the ligand solution, HEPES buffer (25 mM, pH 7.0, with NaCl (150 mM)) was degassed for 10 min and slowly purged with nitrogen for 30 min. This buffer-ligand solution was mixed with a stock solution of 10% (w/v) DDM (final concentration: 0.05% (w/v)) and 20 mM sodium ascorbate (final concentrations: 2 μM−5 mM). The temperature of the sample chamber was maintained at 20 °C with a thermal circulator. Five measurements of the time course of the reduction of oxidized Dcytb with various concentration of ascorbate were measured and averaged. The data were fitted by a combination of four exponential functions using Igor Pro 6 software (Wavemetrics). The rate constants of the fastest phase *k*_1_ (s^−1^) were plotted against the ascorbate concentrations, and the dissociation constant *K*_s_ and rate constant *k*_max_ in the reaction of ascorbate-dependent heme reduction were obtained by Eq. (1).

1 $$k_1 = \frac{{k_{{\mathrm{max}}}{\mathrm{[Ascorbate ]}}}}{{K_{\mathrm s} + [{\mathrm{Ascorbate}}]}}$$

### Resonance Raman spectroscopy

Resonance Raman spectra were recorded with a liquid nitrogen-cooled CCD detector (Roper Scientific, Spec 10:400B/LN) attached to a single polychromator (Jovin Yvon, SPEX750). The 441.6 nm line from a He-Cd laser (Kinmon Electric, model CD4805R) was adjusted to 5 mW at the sample point for excitation. Raman scattering was collected at right angles to the incident light and focused on the entrance slit (150 μm) of the polychromator. Spectra were obtained at ambient temperature with a quartz spinning cell (2000 rpm) having a diameter of 8 mm. Raman shifts were calibrated with indene. The Dcytb samples (final concentration: 20 μM) with various concentrations of ZnCl_2_ (0–10 mM) were prepared in Tris-HCl buffer (50 mM, pH 8.0, containing NaCl (150 mM) and DDM (0.025%)) and were introduced into the Raman cell sealed with a rubber septum. The samples were reduced by the addition of dithionite solution (final concentration: 200 μM) under an N_2_ atmosphere. Electronic absorption spectra were recorded before and after the Raman measurements to confirm that the samples were not damaged by laser irradiation.

Raman spectra were analyzed with Igor software (WaveMetrics). After baseline correction, the spectra were normalized to the intensity of the ν_7_ line at 675 cm^−1^. Raman signals around 365–385 cm^−1^ were deconvoluted as two Gaussian functions. The dissociation constant (*K*_d_) for Zn^2+^ binding to Dcytb was calculated by fitting the titration data to Eq. (2), which assumes the presence of a single binding site for Zn^2+^.

2 $${{S}}_{{\mathrm{obs}}} = {{S}}_0 + \left( {{{S}}_{{\mathrm{max}}} - {{S}}_0} \right) \\ \times \frac{{\left[ {{\mathrm {Dcytb}}} \right] + \left[ {{\mathrm {Zn}}^{2 + }} \right] + {{K}}_{\mathrm{d}} - \sqrt {\left( {\left[ {{\mathrm {Dcytb}}} \right] + \left[ {{\mathrm {Zn}}^{2 + }} \right] + {{K}}_{\mathrm{d}}} \right)^2 - 4\left[ {{\mathrm {Dcytb}}} \right]\left[ {{\mathrm {Zn}}^{2 + }} \right]} }}{{2[{\mathrm {Dcytb}}]}}$$

In this equation, *S*_obs_ denotes the estimated area of the Gaussian signal at 381 cm^−1^, *S*_max_ and *S*_0_ represent the area of the Gaussian peak at 381 cm^−1^ for Zn-free and Zn-bound samples, respectively, and [Dcytb] and [Zn^2+^] denote the total concentrations of Dcytb and Zn^2+^ in the sample solution, respectively.

### Ferric reductase activity assay

*Saccharomyces cerevisiae hem1*Δ*fre1*Δ*fre2*Δ YPH499 strain (*hem1*::KanMX *fre1*::LEU2 *fre2*::HIS3)^47^ was used for the Fe^3+^ reductase activity assay. Because yeast gene *hem1* encodes ALA synthase that catalyzes synthesis of ALA, the first step of heme biosynthesis, all growth media were supplemented with 250 μM ALA to assure adequate heme synthesis for production of Dcytb. Fe^3+^ reductase activity exhibited by this transformed yeast mutant was entirely attributable to expression of the enzyme from the high copy-number plasmid pYES-DEST52 (Thermo Fisher Scientific) carrying the yeast codon-optimized human *Dcytb* gene. Yeast transformation and selection were initially performed following growth on appropriate synthetic complete (SC) medium supplemented with raffinose (2% w/v) and ALA (250 μM). The resulting cells were suspended and grown in SC medium containing raffinose (2% w/v), ALA (250 μM), and galactose (0.4% w/v) for induction of Dcytb expression or glucose (0.4% w/v) for suppression of Dcytb expression at 30 °C for 24 h and subjected to Fe^3+^ activity assay^48^. Dcytb expression was evaluated by western blot analysis (Supplementary Fig. 7) with the anti-human Dcytb mouse monoclonal antibody (homemade, clone 11–2.1). Cells were washed with washing buffer (BSA (2% w/v), Tween 20 (0.1% w/v) in 2× PBS) and then washed twice with reaction buffer (glycerol (5% w/v) and sodium citrate buffer (50 mM, pH 6.5)) or Tris/HCl buffer (40 mM, pH 8.0). Cells suspended in reaction buffer were distributed into 96-well plates, and A_600nm_ was measured with a plate reader (SpectraMax 190, Molecular Devices). An equal volume of assay buffer (reaction buffer containing bathophenanthroline disulfonate (2 mM) and FeCl_3_ (2 mM)) was added to the cells (*T* = 0) and incubated in the dark at 30 °C, 225 rpm until a red color developed. Absorbance values at 535 and 610 nm were determined, and Fe^3+^ reductase activity (nmol 10^6^ cells^−1^ h^-1^) was calculated with Eq. (3).

3 $$\frac{{\left[ {\left( {{\mathrm A}_{535\,{\mathrm {nm}} ({\mathrm {sample}})} - {\mathrm A}_{610\,{\mathrm {nm}}({\mathrm {sample}})}} \right) - \left( {{\mathrm A}_{535\,{\mathrm {nm}}({\mathrm {blank}})} - {\mathrm A}_{610\,{\mathrm {nm}}({\mathrm {blank}})}} \right)} \right] \times 45}}{{V_{{\mathrm {cells}}} \times \left( {{\mathrm A}_{600\,{\mathrm {nm}}({\mathrm {sample}})} - {\mathrm A}_{600\,{\mathrm {nm}}({\mathrm {blank}})}} \right) \times T_{{\mathrm {hour}}}}}$$

The resulting values of Fe^3+^ reductase activity were divided by the amount of Dcytb, wild-type or variant proteins, in each reaction mixture as estimated from the areas of the bands observed on western blots with ImageJ software^49^. To examine the competitive binding assay between Fe^3+^ and Zn^2+^, 0.00, 0.25, 0.50, 0.75, and 1.00 mM ZnSO_4_ was added to the assay buffer containing 2 mM FeCl_3_. To investigate the effects of dietary metal-chelators, the Fe^3+^ activity assay was performed in MES buffer (50 mM, pH 6.5) and Tris buffer (20 mM, pH8.0) with the assay buffer containing 2 mM chelator (sodium citrate, citric acid, malic acid, oxalic acid, glucose, or fructose) and 2 mM FeCl_3_.

### Data availability

The atomic coordinates and structure factors for Dcytb (PDB IDs: 5ZLE for the substrate-free form and 5ZLG for the Zn^2+^ and ascorbate-bound form) have been deposited in the PDB (http://www.wwpdb.org). X-ray diffraction images have also been deposited in SBGrid Data Bank (https://data.sbgrid.org) as IDs 573 and 574, respectively. Data of stopped-flow kinetics have been deposited in Open Science Framework (10.17605/osf.io/4jkr9).

## Electronic supplementary material

Supplementary Information
