# Supplementary material for: Structural basis for promotion of duodenal iron absorption by enteric ferric reductase with ascorbate
Source: Commun Biol. 2018 Aug 17;1:120. doi: 10.1038/s42003-018-0121-8 (PMC6123691; doi:10.1038/s42003-018-0121-8)
Supplement: Supplementary file 1 — Supplementary Information [file 42003_2018_121_MOESM1_ESM.pdf]

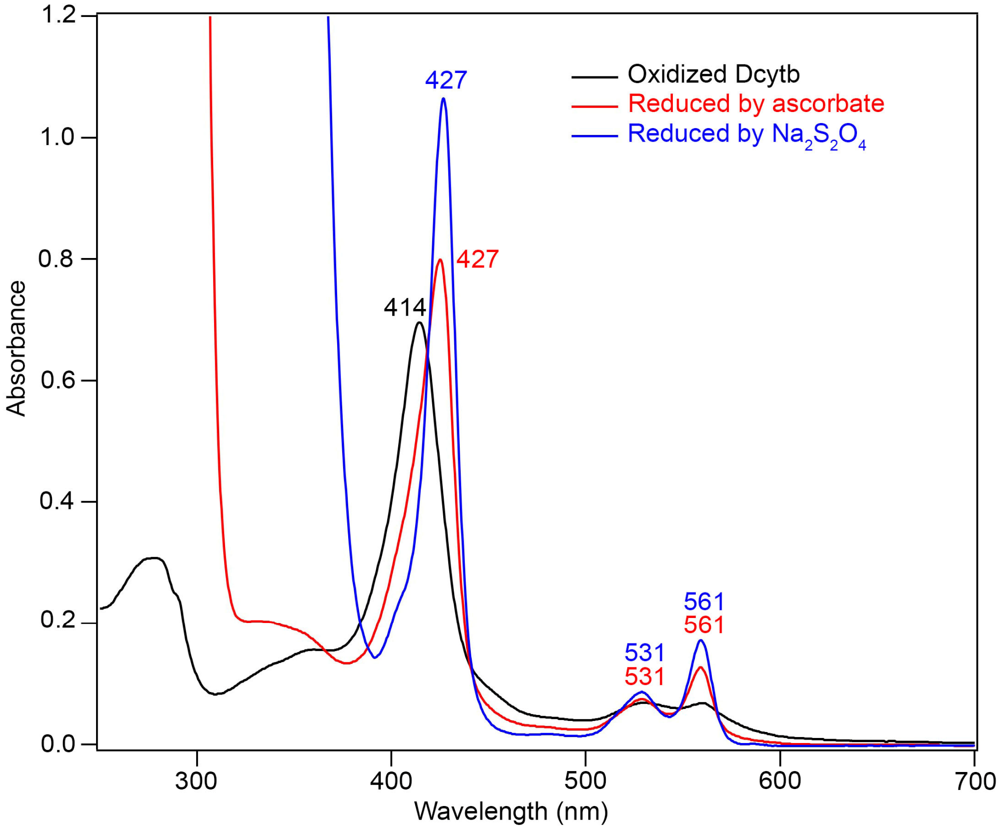

Supplementary Fig. 1. Electronic absorption spectra of oxidized and reduced Dcytb. The spectrum of Dcytb with ferriheme (black) is shown with the spectrum of Dcytb with ferroheme reduced by ascorbate (red) and dithionite (blue). The spectrum was measured in MES-Na buffer (50 mM, pH 6.5) containing NaCl (150 mM) and DDM (0.025 %) at room temperature.

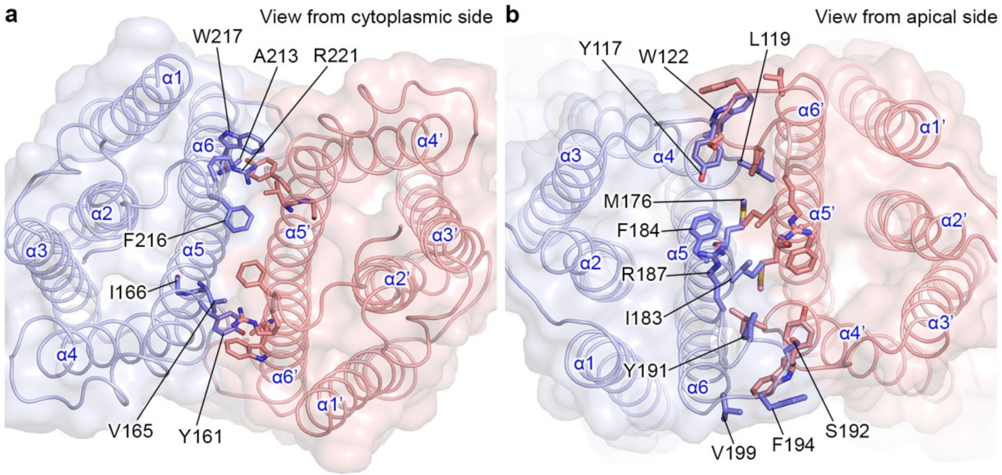

Supplementary Fig. 2. Homodimeric structure of Dcytb. Residues from each monomer (shown in light blue and salmon) associate with each other through hydrophobic interactions. **a.** View from the cytoplasmic side. Hydrophobic interactions involve residues from α-helices 5 and 6 of each monomer. **b.** View from the apical side. Hydrophobic interactions involve residues from α-helices 4, 5 and 6 of each monomer.

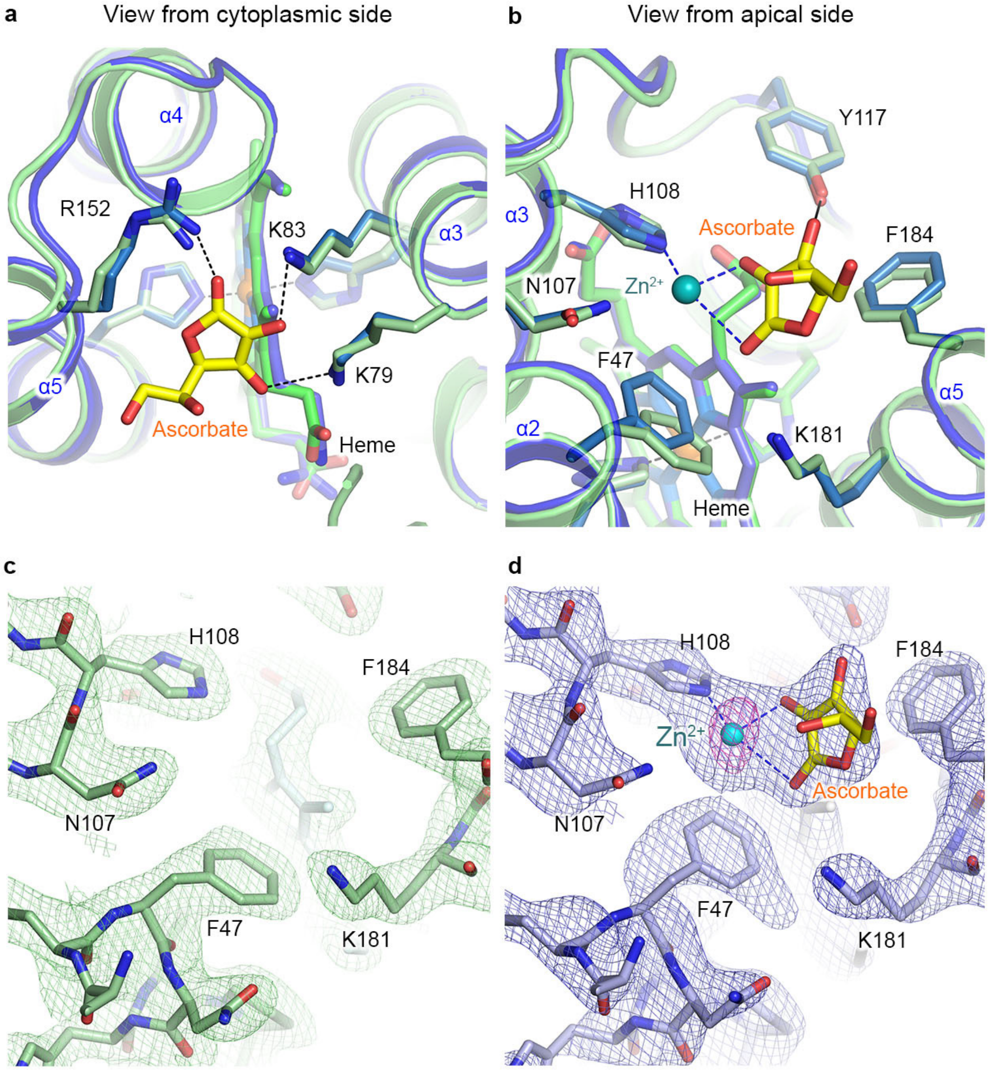

Supplementary Fig. 3. Superposition of apo- and holo- Dcytb. a. Close-up view of the superposition from the cytoplasmic side. Residues that interact with ascorbate are shown as sticks. **b.** Close-up view of the superposition from apical side. Residues that coordinate Zn^{2+}-ascorbate are shown as sticks. Of the amino acid residues in this vicinity, only F47 exhibits any change in orientation upon Zn^{2+}-ascorbate binding. **c.** Electron density map of residues around apical side of apo-Dcytb, contoured at 1.5σ. **d.** Electron density map of residues around apical side of holo-Dcytb, contoured at 1.5σ. Density of F47 changed upon Zn^{2+}-ascorbate binding.

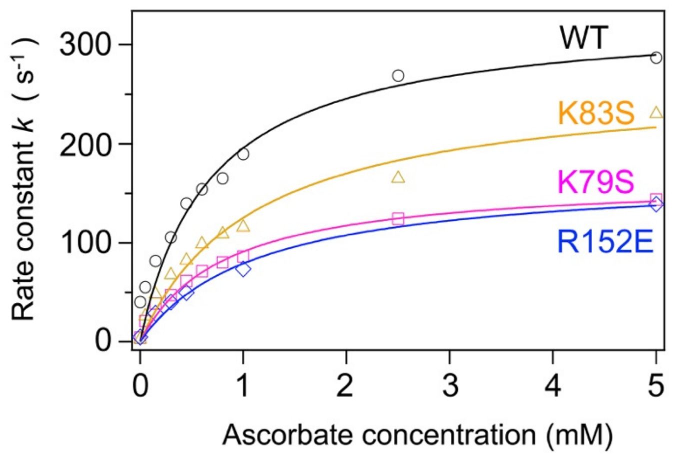

Supplementary Fig. 4. Dependence of the initial phase of heme reduction of wild-type and variant forms of Dcytb on [Ascorbate]. In comparison to the wild-type enzyme, the rates of K79S and R152E reduction are nearly halved while the rate of K83S reduction is reduced by ~20% (Supplementary Table 1).

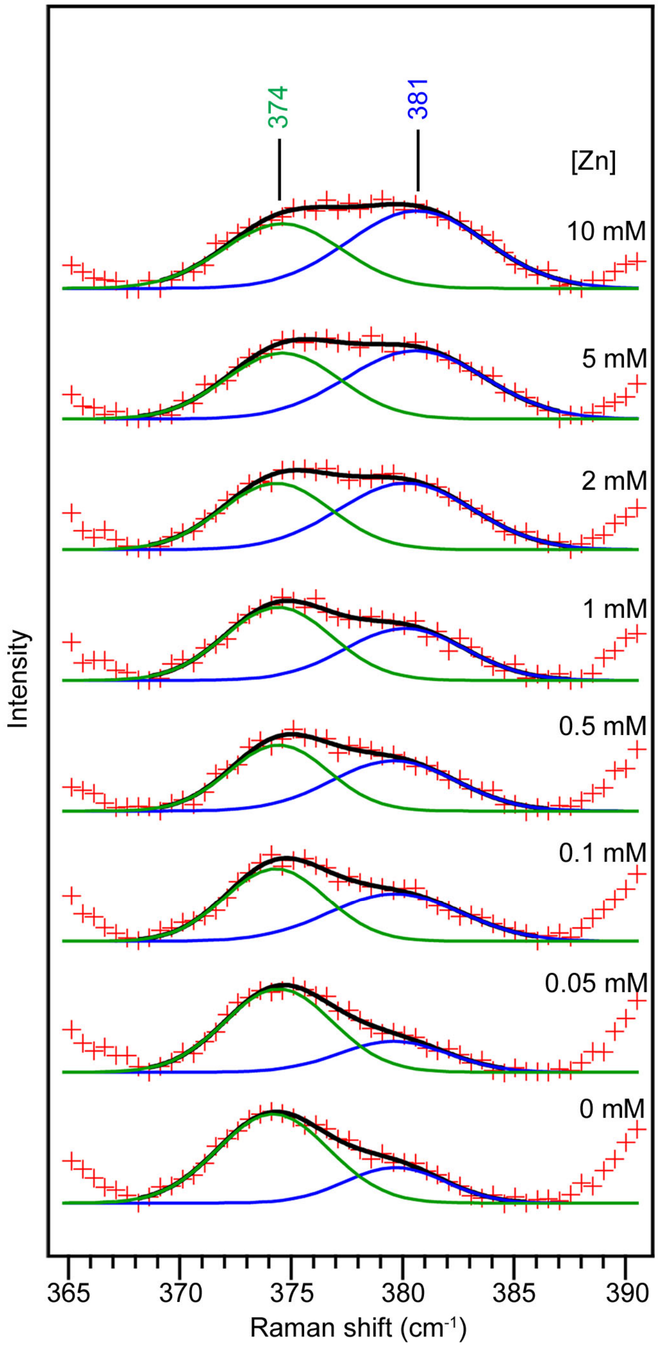

Supplementary Fig. 5. Titration of Dcytb with Zn^{2+} as monitored by resonance Raman spectroscopy. The resonance Raman spectra of reduced Dcytb in the presence of various concentrations of Zn^{2+} (red symbols) were obtained with 441.6 nm excitation. The Raman signals around 365~385 cm^{-1} were deconvoluted as two Gaussian functions. Black traces represent the fit of these functions to the data. The Gaussian components at 374 cm^{-1} (green) and 381 cm^{-1} (blue) are shown.

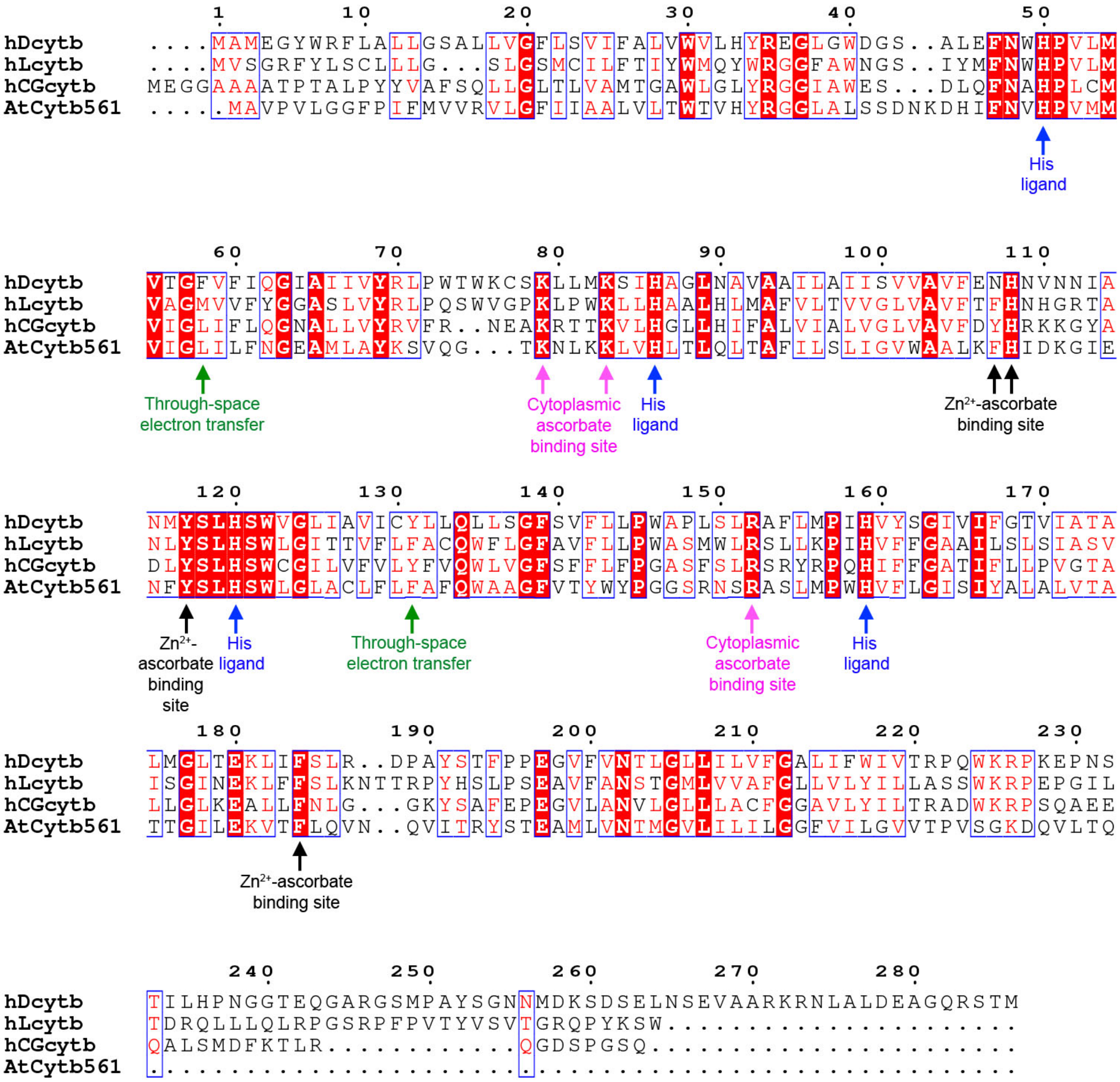

Supplementary Fig. 6. Sequence alignment of Dcytb with *At*Cytb$_{561}$ and other human members of Cytb$_{561}$ family. The four sequences shown here are duodenal Cytb (Dcytb), late-endosomal Cytb (Lcytb), chromaffin granule Cytb (CGcytb) from *Homo sapiens*, and Cytb$_{561}$ from *Arabidopsis thaliana* (*At*Cytb$_{561}$). Residues conserved in all four sequences are highlighted in red. Dcytb has a C-terminal sequence that is longer than all other members of this family.

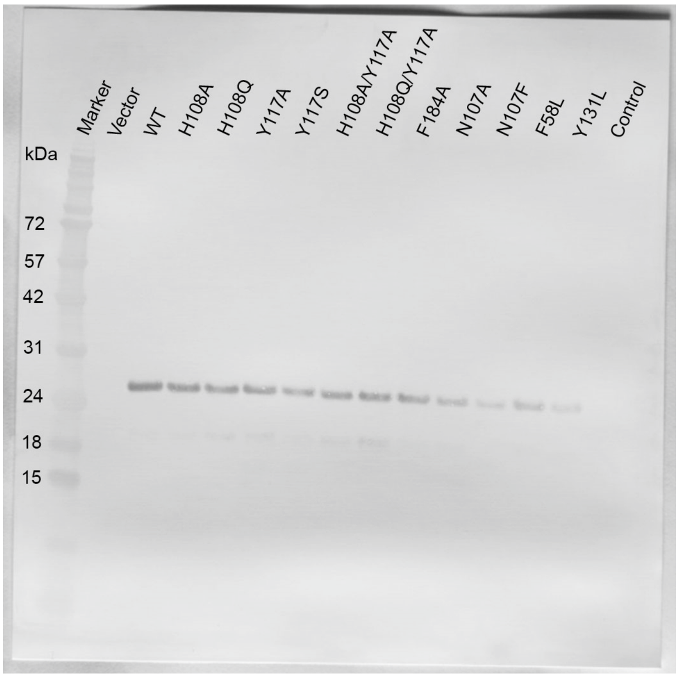

Supplementary Fig. 7. Western blot analysis of whole cell lysate of Dcytb-expressed yeasts. Uncropped membrane of western blot from Fig. 5b is shown. 1.0 μg protein was loaded per lane, and expressed protein was detected by anti-human Dcytb mouse monoclonal antibody (homemade, clone 11-2.1)

Supplementary Table 1. Comparison of dissociation constant and rate constant in the reaction of ascorbate-dependent heme reduction.

	K_s (mM)	k_{max} (s^{-1})
Wild type	0.68±0.07	330±10
K79S	0.81±0.05	165±4
K83S	1.1±0.2	270±20
R152E	1.1±0.2	170±10
